# The Genome Landscape of the African Green Monkey Kidney-Derived Vero Cell Line

**DOI:** 10.1093/dnares/dsu029

**Published:** 2014-09-28

**Authors:** Naoki Osada, Arihiro Kohara, Toshiyuki Yamaji, Noriko Hirayama, Fumio Kasai, Tsuyoshi Sekizuka, Makoto Kuroda, Kentaro Hanada

**Affiliations:** 1 Division of Evolutionary Genetics, Department of Population Genetics, National Institute of Genetics, Mishima, Shizuoka 411-8540, Japan; 2 Laboratory of Cell Cultures, National Institute of Biomedical Innovation, Ibaraki, Osaka 567-0085, Japan; 3 Department of Biochemistry and Cell Biology, National Institute of Infectious Diseases, Tokyo 162-8640, Japan; 4 Pathogen Genomics Center, National Institute of Infectious Diseases, Tokyo 162-8640, Japan

**Keywords:** Vero cell, whole genome, infectious diseases, vaccine, animal cell substrate

## Abstract

Continuous cell lines that originate from mammalian tissues serve as not only invaluable tools for life sciences, but also important animal cell substrates for the production of various types of biological pharmaceuticals. Vero cells are susceptible to various types of microbes and toxins and have widely contributed to not only microbiology, but also the production of vaccines for human use. We here showed the genome landscape of a Vero cell line, in which 25,877 putative protein-coding genes were identified in the 2.97-Gb genome sequence. A homozygous ∼9-Mb deletion on chromosome 12 caused the loss of the type I interferon gene cluster and cyclin-dependent kinase inhibitor genes in Vero cells. In addition, an ∼59-Mb loss of heterozygosity around this deleted region suggested that the homozygosity of the deletion was established by a large-scale conversion. Moreover, a genomic analysis of Vero cells revealed a female *Chlorocebus sabaeus* origin and proviral variations of the endogenous simian type D retrovirus. These results revealed the genomic basis for the non-tumourigenic permanent Vero cell lineage susceptible to various pathogens and will be useful for generating new sub-lines and developing new tools in the quality control of Vero cells.

## 1. Introduction

Continuous cell lines that originate from mammalian tissues serve as not only invaluable tools for life sciences, but also important animal cell substrates for the production of various types of biological pharmaceuticals. One lineage of the most frequently utilized mammalian cell lines for these purposes is the Vero cell lineage, which was established from the kidney tissue of an African green monkey (AGM). The primary culture of this tissue was started on 27 March 1962 in Chiba University in Japan, several continuous cell sub-lines were obtained after passages for several months, and a sub-line was then chosen as the standard Vero cell line.^1,2^

Vero cells were found to be highly susceptible to various types of viruses including simian polyoma virus SV-40,^1,2^ measles virus,^3^ rubella virus,^4,5^ arboviruses,^6,7^ and adenoviruses^7^ soon after their establishment, and were later found to be also susceptible to bacterial toxins including the diphtheria toxin,^8^ heat-labile enterotoxins,^9^ and Shiga-like toxins (or ‘Vero’ toxins).^10,11^ After their global distribution,^12,13^ the application range of Vero cells extended from virology in academic laboratories to diagnostic practices in hospitals and bacterial toxin assays. Vero cells have pseudo-diploid karyotypes^14^ and are non-tumourigenic when a cell passage was not prolonged.^15,16^ Therefore, the Vero cell lineage has been successfully utilized as a cell substrate for human vaccines.^17,18^ Vero cells are still the first choice cell model for various types of life-threating emerging pathogens such as H5N1 influenza virus,^19^ Ebola haemorrhagic fever virus,^20^ and middle east respiratory syndrome (MERS) coronavirus.^21^ Animal substrates for vaccine manufacturing are desired to be shifted from animals and eggs to assured continuous cell lines, because animal materials have several concerns related to quality control, stable supply, and animal ethics.^17–19^ In addition, antigenic drift affecting the vaccination efficacy to humans, which often occurs during the proliferation of influenza viruses in hen eggs,^22^ might be improved by shifting to vaccine strain-susceptible human or non-human primate culture cells.

Therefore, the Vero cell lineage should be fully characterized using modern technologies to prepare threats of infectious diseases. The whole-genome sequences of continuous cell lines provide invaluable basic information for various purposes. The genome sequence of a cell line is a comprehensive basis for many genetic characteristics of the cell line, it is closely relevant to other omics approaches such as transcriptomics and proteomics on the cell line, and also facilitates targeted genome editing of the cell line.^23–25^ However, the whole genome of Vero cells has not yet been determined. We here provided a draft sequence of the whole genome of the Vero cell lineage after massively parallel sequencing of the genome DNA and also karyological and RNA-seq analyses. The genome landscape gave a mechanical insight into events that had occurred during the establishment of the permanent cell line susceptible to various types of microbes. In addition, we presented a proof of concept for the genomic-based quality control of cell lines.

## 2. Materials and methods

### 2.1. Karyological analysis

Metaphase chromosomes from Vero cells and AGM peripheral blood mononuclear cells (PBMC) were analysed by conventional Giemsa-banding (G-banding)^26^ and multi-colour fluorescence *in situ* hybridization (M-FISH) with 24 differentially labelled human chromosome-specific painting probes (24xCyte kit MetaSystems, Altlussheim, Germany). For detailed information, see Supplementary data.

### 2.2. Genome DNA preparation and de novo assembly

Genome DNA was prepared from Vero cells (with passage number 115) and PBMC using the Qiagen Blood & Cell Culture DNA kit (Qiagen GmbH, Hilden, Germany). Libraries constructed for paired ends and mate pairs were sequenced with HiSeq2,000 (Illumina Inc., San Diego, California). After quality filtering, sequences were assembled into scaffolds using SGA and SSPACE software^27,28^ (see Supplementary data for detailed assembly procedure). Protein-coding genes were predicted by the AUGUSTUS program with reference to the human genome as a model^29^ and also with RNA-seq reads to assist in the predictions.

### 2.3. Mapping to the rhesus macaque and AGM reference genome

Reads were mapped on the draft genome of the rhesus macaque (*Macaca mulatta*: rheMac2) and AGM (*Chlorocebus sabaeus* 1.0: GCA_000409795.1) using the BWA-MEM algorithm with default parameter settings.^30^ After mapping, potential polymerase chain reaction (PCR) duplicates, which were mapped to the same positions of the reference genome, were removed using Picard software (http://picard.sourceforge.net). The average genome coverage of paired-end sequences after removing the PCR duplicates was 54-fold for the AGM reference. Single-nucleotide variants (SNVs) were called following the Best Practice pipeline of the Genome Analysis Toolkit (GATK) software package, which includes base quality score recalibration, insertion/deletion (indel) realignment, and discovering and filtering SNVs and indels.^31^

### 2.4. Detection of genomic rearrangements in the Vero JCRB0111 cell line

Copy number variants were detected using the Control-FREEC software^32^ with a 100-kb window size and 20-kb step size. Sites with map quality scores <40 were not used in the analysis. Structural variants were identified using the integrated structural variant prediction method DELLY. Junction sequences with ≥85% identity to the other part of the reference genome and split-read coverage >100 were also filtered out.

To reduce rare and false-positive variant calls, we further applied the following conservative criteria. To detect deletions and inversions, we counted reads spanning non-rearranged sequence regions with at least 7 bp overlapping to each sequence proximal and distal to the boundaries. The number of these canonical reads should be proportional to the number of non-rearranged cells. The number of canonical reads was calculated for each non-rearranged region and divided by 2, because one rearrangement had two non-rearranged regions. We selected the regions at which rearranged reads (split reads) consisted of at least 70% of total reads mapped on boundary regions (sum of canonical and split reads). We also filtered out the regions that had <20 paired-end supports. For additional information, see Supplementary data.

Loss-of-heterozygosity (LOH) regions were identified using 1-Mb-size windows with average heterozygosity <0.0005 and the ratio of homozygous to heterozygous SNVs smaller than 0.2. The cut-off criteria were determined using the distribution of these values in a whole genome (Supplementary Fig. S3). The windows were progressively merged into larger regions when average statistics in the region satisfied the criteria.

### 2.5. Miscellaneous

Procedures for cell culture, tumourigenicity test, RNA-seq, phylogenetic analysis, and genomic PCR are described in Supplementary data.

### 2.6. Ethics

All animal experimental procedures were approved by the National Institute of Biomedical Innovation Committee on Animal Resources as the Institutional Animal Care and Use Committee.

### 2.7. Accession codes

The short reads and assembled draft genome sequence have been deposited in the public database (accession number: DRA002256). The full-length simian endogenous retrovirus sequences obtained in Vero JCRB0111 cells have been deposited in DDBJ (accession number: AB935214).

## 3. Results

### 3.1. Vero cell seed

To obtain the reference genome sequence of the cell lineage, cell seeds with the least passage levels were desirable as material. We chose a cryopreserved cell lot registered at the Japanese Collection of Research Bioresources Cell Bank, which, to the best of our knowledge, is the oldest or nearly the oldest lot (with a passage level of 115 from the original primary culture started in March 1962) among the currently available stocks. Lot JCRB0111 (hereafter referred to as Vero JCRB0111) was expected to be a close relative to widely distributed Vero cell seeds such as ATCC CCL81 and WHO Vero 10–87.^1,2,12,13,33–35^ The heteroplantation of Vero JCRB0111 cells in immunocompromised nude mice (*n* = 10) did not produce any discernible tumours, while that of human cervical carcinoma-derived HeLa cells produced tumours in nude mice at 100% efficacy. Thus, Vero JCRB0111 cells are non-tumourigenic, which is consistent with previous findings of Vero cells being non-tumourigenic when their passage number was limited.^15,16^

When choosing the Vero JCRB0111 cell seed in this study, we confirmed that there was no microbial contamination in the seed using conventional tests. In addition, DNA-seq short reads, which were obtained to resolve the draft sequence of the whole Vero cell genome, were employed to comprehensively detect microbe-relevant sequences with a megablast search. No discernible sign of microbe-relevant sequences (except for endogenous retroviral sequences as shown below) was detected in the cell genome sample, which confirmed the absence of microbial contamination in the Vero cell seed.

### 3.2. Karyotyping

**Figure 1.**
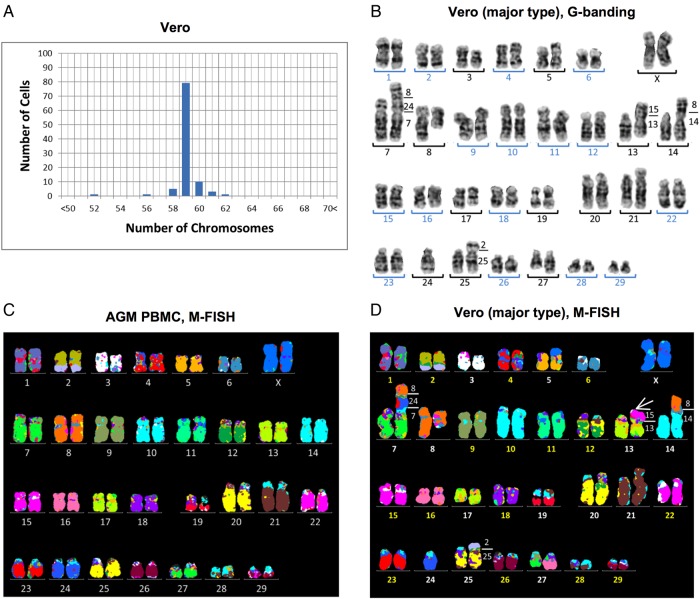
Karyotyping of the Vero JCRB0111 cell line. (A) Chromosome number in the Vero cell line based on 100 Giemsa-stained metaphases, which showed that the modal number was 59 chromosomes and differences in the chromosome number between 52 and 62 indicated heterogeneous karyotypes. (B) G-banded karyotype of Vero cells with 59 chromosomes consisted of 16 homologous pairs (blue numbers) and 13 abnormal chromosomes (black numbers). (C and D) M-FISH signal pattern using human probes on a normal AGM metaphase (C), and a main clone of the Vero cell line (D). Paints of human chromosomes 1–22 and X showed homologous regions in the AGF and the identity of 32 and 40 syntenic blocks in a normal female and Vero, respectively. The number on the right of aberrant chromosomes (D) shows correspondence to AGF chromosomes. The derivative chromosome der(13) (white arrow in D) showed an extra signal that corresponded to AGM chromosomes 15 and 22, both of which were painted by human chromosome 3. Although the addition in der(13) could not be distinguished using human M-FISH probes, the copy number analysis revealed a gain at chromosome 15q (Fig. 2B), which indicated that der(13) had additional material over chromosome 15.

The Vero JCRB0111 cell line had different karyotypes with chromosomes numbering between 52 and 62, and the modal chromosome number appeared to be 59 chromosomes in 79 of 100 metaphase cells (Fig. 1A). The same analysis on PBMC from normal female AGM showed 60 chromosomes (Supplementary Fig. S1A).^36^ Because the G-banding karyotypes of Vero cells include several abnormal chromosomes (Fig. 1B), M-FISH was applied to identify chromosomal rearrangements. Human M-FISH probes hybridized efficiently to AGM chromosomes and showed 32 syntenic blocks in normal AGM karyotypes (Fig. 1C). This homology to humans was consistent with previous findings,^37^ and implied that the human M-FISH signal pattern can be used as a normal AGM reference (Fig. 1C). G-banding and M-FISH analyses revealed that 18 of 37 Vero metaphases represented a main clone with 59 chromosomes. M-FISH identified 40 segments in the Vero metaphases (Fig. 1D), which indicated the occurrence of one fusion and seven translocations. The fusion occurring between chromosomes 7 and 24 led to a reduction in the chromosome number from 60 to 59. Duplications and deletions were also detected in 6 chromosomes (Fig. 1D), showing rearrangements involved in 14 chromosomes. However, major chromosomal rearrangements were not detected in the other 46 chromosomes by G-banding or M-FISH (Fig. 1B and D), which suggested that a haploid chromosome set was retained in its original form. In addition to these common features, additional abnormalities were detected in 11 of 37 cells (Supplementary Fig. S1C and D). Although the other eight karyotypes were found to have similar abnormalities, the Vero cell line appeared to include several subclones. The main clone accounted for less than half of the population (18 of 37 cells) and the Vero cell line had a high cytogenetic heterogeneity.

### 3.3. De novo assembly of the Vero genome sequence

Two paired-end and three different size mate-pair libraries were constructed from Vero cell DNA, and the libraries were sequenced by massively parallel sequencing. The insert size distribution and read length of the libraries are summarized in Supplementary Table S1. After quality filtering, we obtained a total of ∼2.55 billion paired-end and 390 million mate-pair reads. The filtered sequences were used for *de novo* assembly of the Vero genome. Our final scaffolds consisted of 401,905 scaffolds, which were 2.97 Gb in total, and had the N50 of 508 kb and N90 of 48 kb. The AUGUSTUS program identified 25,877 putative protein-coding genes in the genome scaffolds, using RNA-seq data as a support.

### 3.4. The female C. sabaeus origin of the Vero cell lineage

**Figure 2.**
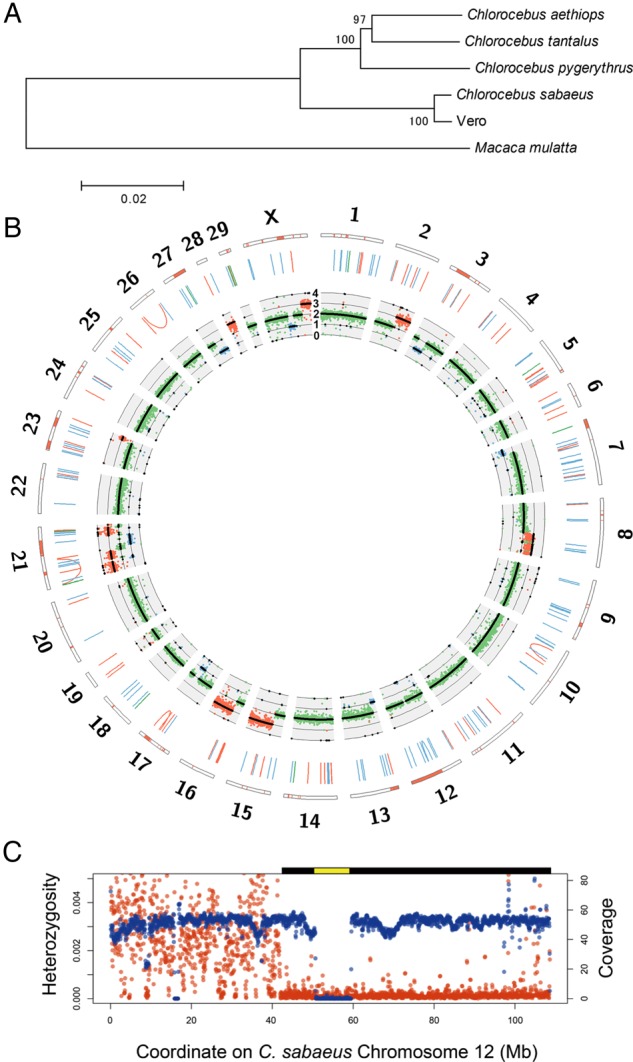
Genome landscape of the Vero genome. (A) Phylogeny of mitochondrial genomes of the Vero cell line, four *Chlorocebus* species, and *Macaca mulatta*. Bootstrap values with 1,000 replications were shown upon the branches. (B) Circos plot of the Vero cell genome. The orange bars in the outermost rectangles represent LOH regions. The blue, green, and orange lines in the middle layer show deletions, duplications, and inversions larger than 1 kb, respectively. The innermost plot shows the coverage of paired-end reads and expected ploidy (black lines). The blue, green, and orange dots represent the coverage values in 1×, 2×, and 3× regions, respectively. (C) The large deletion and LOH regions on *Chlorocebus sabaeus* chromosome 12. The red and blue points represent average heterozygosity (the number of heterozygous SNVs per bp) and genome coverage of paired-end reads in 1-Mb-size windows, respectively. The predicted homozygous deletion regions and LOH regions are shown as yellow and black bars on the plot area, respectively.

The Vero cell lineage was originally reported to be established from the kidney of the AGM *Cercopithecus aethiops*.^1,2^ However, the species classification of AGMs is a still debated issue and has been revised several times.^38,39^ In current nomenclature, *Cercopithecus aethiops* is further classified into four different species of *Chlorocebus*. To clarify the species origin of the Vero cell lineage, we compared our reads with four previously reported complete mitochondrial sequences of *Chlorocebus* species: *Chlorocebus aethiops*, *Chlorocebus tantalus*, *C. sabaeus*, and *Chlorocebus pygerythrus*.^40^ The mutation rate in the mitochondrial genomes of Old World monkeys is known to be markedly higher than that in nuclear genomes^41^; therefore, the mitochondrial sequences of these four species had sufficiently diverged to cause mapping bias of short reads. We mapped the paired-end reads to all four *Chlorocebus* mitochondrial genomes as a reference and found that the mitochondrial genome of *C. sabaeus* had the highest coverage and lowest divergence to the mitochondrial genome of Vero cells (Supplementary Table S2). Furthermore, a phylogenetic tree using whole mitochondrial genome sequences indicated that the Vero cell line was closest to *C. sabaeus* (Fig. 2A). The gender of the AGM individual from which the Vero cell lineage was established has not yet been clearly described. A pair of X chromosomes in karyotyping (Fig. 1) and the almost diploid copy number of X chromosomes in sequence data (Fig. 2B) were observed in Vero JCRB0111 cells. Collectively, we concluded that the Vero cell lineage had been established from a female individual of *C. sabaeus*.

### 3.5. Identification of SNVs

To characterize SNVs in the Vero cell line nuclear genome, we mapped our paired-end reads to the reference genome of the rhesus macaque (*M. mulatta*), which has been annotated more than other non-human primate genome sequences. A total of 92% of the unambiguous sites of the rhesus macaque draft genome was covered with high-quality reads, and the genotype was called with high confidence (genotyping quality score ≥45 and coverage ≥10), which indicated that the rhesus macaque genome would work as a reasonable reference sequence for analysing the Vero cell genome. Approximately 58.5 million SNVs were identified (Supplementary Table S3). Of these, ∼51.2 million and 7.3 million SNVs were homozygous and heterozygous, respectively. Most of the homozygous SNVs in the Vero cell line were attributed to evolutionary divergence between the genus *Macaca* and *Chlorocebus*, which was ∼8–12 million years ago.^42,43^ The estimated divergence between the rhesus macaque and Vero cell line genomes was ∼2.2%. The level of heterozygous SNVs in the Vero cell line was also high and was markedly higher than that in a human individual.^44^ This may be partly explained by the higher genetic diversity within AGM populations. We also identified ∼2.9 and 2.7 million small deletions (≤28 bp) and insertions (≤44 bp), respectively (Supplementary Table S3).

### 3.6. Detection of genomic rearrangements in the Vero cell line

By comparing our paired-end reads with the publicly available draft genome of the AGM *C. sabaeus* (*C. sabaeus* 1.0: GCA_000409795.1), we examined potential >1- kb-scale deletions, segmental duplications, inversions, and translocations using the information of improper paired-end mapping and split-read mapping.^45^ In order to present a landscape of genomic rearrangements in the main population of the Vero cell lines, we set stringent criteria for finding genome rearrangements that could identify rearrangements of high frequency in the cell population. A total of 138 deletions, 78 duplications, and 12 inversions of high frequency were detected (Fig. 2B; Supplementary Table S3), whereas none of the translocation candidates were detected at a high frequency. AGM chromosome 12 harboured a homozygous deletion that spanned across nearly 9 Mb (Supplementary Tables S3 and S4). The region was syntenic to human chromosome 9 and rhesus macaque chromosome 15 (from *MLLT3* to *LINGO2*) and contained ∼40 genes including the type I interferon gene cluster and *CDKN2* genes (Fig. 2B and C; Supplementary Table S4). This homozygous large deletion was validated as given below.

### 3.7. Detection of LOH

We identified copy number variations in different genomic regions using the mapping coverage of paired-end reads. Nine regions in chromosomes 2q, 8q, 15q, 16q, 21, 23q, 28, and Xq had three copies, while eight regions at 3p, 7p, 13p, 17q, 21q, 27q, and Xq were identified as a single copy (Fig. 2B). AGM chromosome 8p showed an intermediate coverage between two and three copies, in which the duplication and translocation of AGM chromosome 8p varied among the clones. These large-scale copy number changes agreed well with the karyotypes examined using M-FISH analysis (Fig. 1D). LOH regions were identified using the density of SNVs and its ratio to the density of homozygous SNVs. In addition to the identified haploid regions, we identified large LOH blocks in chromosomes 6q, 12q, and 23. The LOH on chromosome 12 harboured a large homozygous deletion (Fig. 2C; Supplementary Table S4; see also below).

### 3.8. Validation of large deletions by PCR

**Figure 3.**
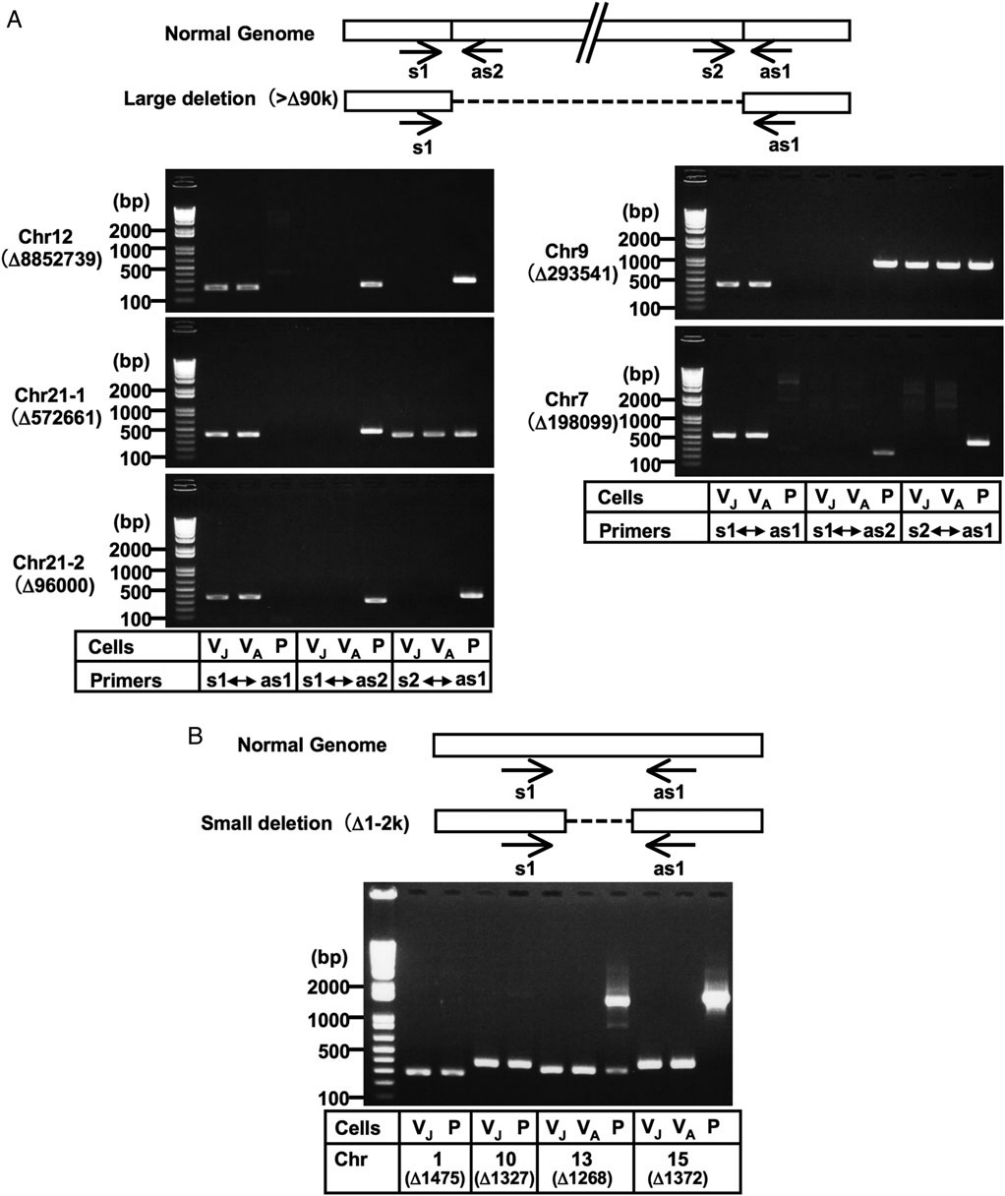
Validation of genomic deletions in Vero cells by PCR analysis. Large deletions (>90 kb deletions) (A) and some small deletions (1–2 kb deletions) (B) were selected. Although the genomic PCR confirmed the existence of breakpoint junctions for the 573 kb deletion in chromosome 21 and the 294 kb deletion in chromosome 9, a part of these regions appeared to exist somewhere in the genome (**A**; see also Supplementary Fig. S2). Amplicons corresponding to the deletions predicted in chromosomes 1 and 10 were produced not only from Vero cells, but also from AGM PBMC, while amplicons corresponding to the ‘non-deleted’ counterparts were not produced even from AGM PBMC (B; see also Supplementary Fig. S2), which indicated that our determined sequences for the Vero cell genome existed homozygously in these regions not only in Vero cells, but also in AGM PBMC. This paradox might be attributed to the possible incompleteness of the currently available version of the AGM whole-genome draft sequence or polymorphic state of the deletion within AGM populations. *Normal Genome* indicates the sequences predicted from the draft genome sequences of AGM and the rhesus macaque. Arrows indicate the primer positions used in the PCR analyses. The ‘Δ’ indicates the genomic deletion size predicted by the massively parallel sequencing system. The templates used were as follows: VJ, Vero JCRB0111; VA, Vero ATCC; P, AGM PBMC. PCR amplicons were sequenced to confirm the breakpoint sequences, which are shown in Supplementary Fig. S2. Chr, chromosome.

The large deletions predicted by the massively parallel sequencing system were validated by genomic PCR. Regarding the 8.85-Mb deletion of chromosome 12, a set of PCR primers striding across the deletion junction produced a ∼230-bp amplicon from Vero cells, but not from normal AGM cells, while a PCR primer set designated for the AGM genome produced a predicted amplicon from normal AGM cells, but not from Vero cells (Fig. 3A). We tested two Vero cell lines, JCRB0111 and ATCC CCL81, and obtained identical results (Fig. 3A). In similar validation tests for another four large (>90 kb) predicted deletions (Supplementary Table S4), DNA fragments with breakpoint junctions were amplified from the Vero cell lines, but not from AGM PBMC for all these deletions, which confirmed the existence of these deletions in Vero cells (Fig. 3A; Supplementary Fig. S2). Four small (1–2 kb) predicted deletions were also confirmed to exist (Fig. 3B; Supplementary Fig. S2).

### 3.9. Proviral SRV in the Vero JCRB0111 genome

**Figure 4.**
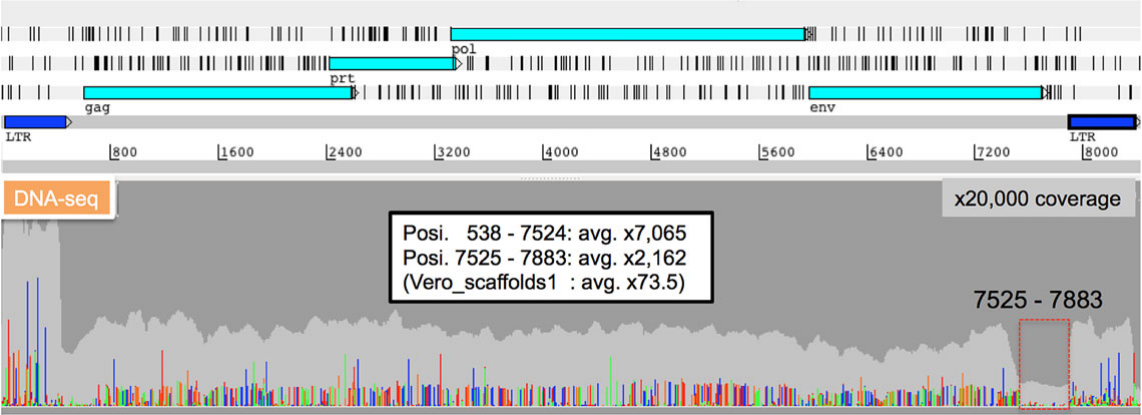
Characterization of proviral SRV sequences. DNA-seq short read mapping to the complete SRV-Vero genome sequence. Read depth and mismatch nucleotides are shown in the following colours (Depth: light grey, A: light green, T: red, G: orange, C: dark blue). The high variability of SRV sequences was rarely detected in the 7525–7883 nt region, whereas high variability was observed throughout other regions.

Analysis of collected SRV-related short reads from all paired-end short reads of the Vero JCRB0111 cell line, followed by analyses of gene assignment and long terminal repeat (LTR) finding, identified the 8,367 bp complete SRV genome sequence. The medians of variant frequencies were 25.5 and 8.0% in the highly variable and *env*-deleted region (nucleotide position 7525–7829) of SRV, respectively (Fig. 4). The copy number of SRV with 8.4-kb full length was estimated to be 30%, while that of SRV with the deletion of the 7525–7883 nucleotide (nt) region encompassing the C-terminal part in *env* and a portion of LTR was 70%. The SRV-Vero of JCRB0111 had 97% of the same nucleotides as those of ATCC CCL81 (Genbank_ID: JN134185). The number of minor alleles was 703 for SNVs and 1 for the insertion among the whole complete consensus SRV sequence in the Vero JCRB0111 genome. Four minor mutation sites caused three nonsense mutations and one frameshift on the *pol* or *env* region (Supplementary Table S5). Previous studies suggested a frameshift mutation in *pol* (position 3726) or frameshift mutation in *prt* (its position was not reported) on the SRV proviral sequences of Vero E6 or ATCC CCL81 cells.^46,47^ However, our study did not detect equivalent mutations on the SRV proviral sequences of Vero JCRB0111 cells; however, other notable mutations were instead detected (Supplementary Table S5).

## 4. Discussion

By comparing with the AGM reference genome, the whole genome structure of Vero cells provided various important insights into the molecular characterization of this cell line. Vero cells are incapable of producing type I interferon in response to viral infections,^48^ which may be the main cause for the high susceptibility of these cells to various types of microbes. The homozygous deletion of α- and β1-interferon genes in Vero cells was previously reported using classical DNA hybridization analysis.^49^ The present study determined an ∼9- Mb deleted region in chromosome 12 at the nucleotide level and further revealed an ∼59-Mb LOH around the deleted region (Fig. 2B and C), which suggested that an ∼9-Mb deletion first occurred in one of two homologous chromosome 12 during the establishment of cells, followed by a large-scale conversion that fixed the homozygous deletion of the region in the Vero cell lineage. The deleted region in chromosome 12 of Vero cells is syntenic to human chromosome 9p21–p22, which contains many genes of type I interferons (α8, α2, α1/13, α6, α14, α4, α17, α21, ω1, and β1) (Supplementary Table S4).

The human syntenic region corresponding to the deleted region in AGM chromosome 12 also contained *CDKN2A* and *CDKN2B*: *CDKN2A* encodes cyclin-dependent kinase (CDK) inhibitor 2A/p16^INK4A^ (which inhibits CDK6, a negative regulator of the retinoblastoma protein pRB) and p14^ARF^ (which inhibits the p53-negative regulator MDM2) in an alternate reading frame to the former, while *CDKN2B* encodes CDK inhibitor 2B/p15^INK4B^ (which inhibits another pRB-negative regulator CDK4) (Supplementary Table S4).^50,51^ The CDK inhibitors and MDM2 inhibitor act as key regulators of the cell cycle, and mutations in *CDKN2A*-*CDKN2B* often occur in various types of human cancer; however, these mutations by themselves are not enough to transform cells into tumourigenic cells.^50–52^ The loss of both *CDKN2A* and *CDKN2B* may play a crucial role in the acquirement of immortality in the Vero cell lineage. Originally non-tumourigenic Vero cells may then acquire tumourigenicity when additional unknown mutations accumulate during prolonged passages.^15^

Although the karyological analysis demonstrated that Vero cells had various chromosomal rearrangements (Fig. 1C), no translocation was identified in the whole-genome sequence (Fig. 2B). This discrepancy may have been due to technical limitations. The karyotyping results obtained showed that most of the translocation events occurred between the telomeric regions of chromosomes, which could not be identified by sequencing if chromosomes fused via repeat sequences. In addition, in order to filter out rare chromosomal rearrangements in a cell population, events that occurred in only one of the homologous chromosomes may not be identified in our filtering criteria. Therefore, the absence of translocation rearrangements by sequencing does not contradict the results of the karyological analysis, which showed that all or most chromosomal translocations were observed in one of the two homologous chromosomes (Fig. 1C and D; Supplementary Fig. S1C and D). Haplotype sequencing may be necessary to determine such heterozygous events.

Many SRV sequence variations existed in Vero JCRB0111 cells (Fig. 4). As for SRV associated with the vaccine-producing Vero E6 cell line (the parental cell line of which is ATCC CCL81), a frame-shifting single-nucleotide insertion in the polymerase gene was identified.^46^ This frameshifting mutation was not detected in SRV associated with the Vero ATCC CCL81 cell line^47^ or Vero JCRB0111 cell line (this study). SRV variant sequences lacking the U3 and R regions of 3′LTR were instead detected in Vero ATCC CCL81-associated SRV,^47^ while the results of this study suggested that some SRV copies in the Vero JCRB0111 cell genome were defective in the *env*-3′LTR region (Fig. 4). Thus, a large amount of diversity may occur in proviral SRV sequences during the passage of Vero cells.

Various quality tests must be conducted in order to fully characterize cell banks for pharmaceutical use^53^ (The WHO guidelines on animal cell substrates are available at http://www.who.int/biologicals/vaccines/TRS_978_Annex_3.pdf.) Many of these tests rely on conventional methodology, and some tests still use many experimental animals. The whole-genome sequence should be invaluable reference information to develop more rational and effective methods for identification, genetic stability, and microbial agents in pharmaceutical cell banks. For example, the currently authorized tests for cell identity consist of classical methods (e.g. isoenzyme analysis and G-band analysis) and more modern DNA profiling methods (e.g. restriction fragment length polymorphism and variable number of tandem repeats analysis). These tests, even if not all, require a considerable amount of time and money as well as well-trained technical skills, and some are not accurate enough to discriminate different cell lines established from the same biological species. This study presented a proof of the concept that PCR analysis will open a rapid and accurate alternative method for the cell identity test to detect unique chromosomal deletions (Fig. 3). Metagenome analysis is a powerful approach that can be used to survey microbial contamination in biological pharmaceuticals.^46,47,54^ This study also employed DNA-seq short reads of Vero cell genome DNA to comprehensively survey microbe-related sequences in the cell sample, and detected no discernible sign of microbe-relevant sequences (except for endogenous SRV) in Vero JCRB0111 cells. In this direction, it is crucial to distinguish between endogenous and exogenous viral-like sequences, because the appearance of the former is inevitable and can serve as an internal positive control in metagenomic analysis for microbial agents. In addition, the heterogeneity in SRV sequences as discussed above may also be a good genomic signature for identifying a specific cell seed among various Vero cell sub-lines. The whole-genome sequence of Vero cells will also be an invaluable resource for engineering the specific genes of cells by recently advanced genome-editing technologies.

In conclusion, this study showed the genomic characteristics of Vero cells, which have been a good cell model for microbial infection for a long time. In addition, the genome landscape will be a crucial resource not only for the quality control of Vero cell lines, but also for the development of novel sub-lines in the future.

## Authors’ contributions

Overall planning: K.H.; design and performing experiments: N.O., A.K., T.Y., and M.K.; data analysis: N.O., A.K., T.Y., N.H., F.K., T.S., M.K., and K.H.; manuscript writing: N.O., A.K., T.Y., T.S., M.K., and K.H. All authors read and approved the manuscript.

## Supplementary data

Supplementary Data are available at www.dnaresearch.oxfordjournals.org.

## Funding

This work was supported by the Japan Society for the Promotion of Science KAKENHI Grant numbers 22370054 and 25670065 (to K.H.) and also by Takeda Science Foundation (to K.H.). Funding to pay the Open Access publication charges for this article was provided by a Grant-in-Aid of Takeda Science Foundation (to K.H.).

## Supplementary Material

Supplementary Data
