# Supplementary material for: The Genome Landscape of the African Green Monkey Kidney-Derived Vero Cell Line
Source: DNA Res. 2014 Sep 28;21(6):673–83. doi: 10.1093/dnares/dsu029 (PMC4263300; doi:10.1093/dnares/dsu029)
Supplement: Supplementary Data [file supp_21_6_673__index.html]

The Genome Landscape of the African Green Monkey Kidney-Derived Vero Cell Line — The Genome Landscape of the African Green Monkey Kidney-Derived Vero Cell Line — Supplementary Data 

# The Genome Landscape of the African Green Monkey Kidney-Derived Vero Cell Line

## Supplementary Data

Supplementary Data

**Files in this Data Supplement:**

- Supplementary Data - Pdf file
