# Supplementary material for: The Genome Landscape of the African Green Monkey Kidney-Derived Vero Cell Line
Source: DNA Res. 2014 Sep 28;21(6):673–83. doi: 10.1093/dnares/dsu029 (PMC4263300; doi:10.1093/dnares/dsu029)
Supplement: Supplementary Data [file supp_dsu029_dsu029supp.pdf]

Supplementary Information to:

The genome landscape of the African green monkey kidney-derived Vero cell line

Table of Content

Supplementary materials and methods. -- 2

References associate with supplementary materials and methods. -------------------------------------- 7

Legends to supplementary figures. -- 9

Table S1. Summary of genome-sequencing libraries of Vero JCRB0111 cells. ------------------------ 10

Table S2. Comparison of mitochondrial genome sequences between the Vero cell line and *Chlorocebus* species. -- 11

Table S3. Number of SNVs and small indels in the Vero cell line genome using the rhesus macaque genome sequence as a reference. -- 12

Table S4. Summary of large deletions in the Vero JCRB0111 cell genome. ---------------------------- 13

Table S5. Single nucleotide mutation and insertion position and mix population rate of the truncated SRV provirus sequence in Vero JCRB0111. -- 15

Table S6. PCR primers used to validate deletions. -- 16

Figure S1. Karyotyping of AGM PBMC and Vero cells. -- 17

Figure S2. Sequence analyses of genomic deletions in Vero cells. -------------------------------------- 18

Figure S3. Distribution of heterozygous SNV density and the homozygous to heterozygous SNV ratio in the 1Mb-size window. -- 23

Supplementary materials and methods

Cell culture

The peripheral blood mononuclear cells (PBMC) of female African green monkeys (AGMs) were obtained from the Tsukuba Primate Research Center of National Biomedical Innovation and cultured in RPMI-1640 medium (Life Technology, Grand Island, NY) supplemented with 4% phytohaemagglutinin-M (Life Technology, Grand Island, NY) and 200 U/ml IL-2 (Sigma-Aldrich, St. Louis, MO). Vero JCRB0111 cells and Vero ATCC (CCL81) cells were maintained in Eagle's minimal essential medium (Life Technology, Grand Island, NY). Both media had 10% heat-inactivated (56°C for 30 min) fetal bovine serum (Sigma-Aldrich, St. Louis, MO). Cells were cultured at 37°C under an atmosphere of 5% CO_2 and 100% humidity.

Tumorigenicity test

Tumorigenicity was tested by injecting 1×10^7 cells subcutaneously into BALB/cAJcl-nu female mice (Nihon Clea, Japan) at 6 weeks of age and measuring the sizes of the resulting tumors at least once a week for 12 weeks. Animal protocols were approved by the committee for the Ethics of Animal Experimentation and were in accordance with the Guidelines for Animal Experiments in the National Institute Biomedical Innovation.

Karyological analysis

Metaphase chromosomes from Vero cells and PBMC were harvested after incubation with 0.05 μg/ml Metaphase Arresting Solution (Genial Genetics, Chester, UK) at 37°C for 4 hours, followed by treatment with a hypotonic solution (75 mM KCl) for 30 min and three successive changes of the fixative solution (methanol/acetic acid, 3:1 by vol./vol.). The nuclear suspensions were dropped onto clean slides and aged at 85°C for 45 min prior to the Giemsa-banding

(G-banding) and multi-color fluorescence *in situ* hybridization (M-FISH) experiments. M-FISH using 24 differentially labeled human chromosome-specific painting probes (24xCyte kit MetaSystems, Altlussheim, Germany) was performed according to the manufacturer's protocol. Briefly, the slides were incubated at 70°C in saline solution (2x saline sodium citrate buffer) for 30 min, denatured in 0.07M NaOH for 1 min, dehydrated in ethanol series, air-dried, covered with 10 μl of probe cocktail (denatured), and hybridized for 4 days at 37°C. After post-hybridization washing at 72°C, the slides were dehydrated in ethanol series and counter-stained with 10 μl of 4′,6-diamidino-2-phenylindole (DAPI)/antifade. The signals were captured and analyzed by the Metafer system and MetaSystems' ISIS software (software for spectral karyotypes).

De Novo assembly

A custom service for the construction of DNA libraries for paired ends with the TruSeq DNA Sample Prep Kit (Illumina Inc.) was provided by Hokkaido System Science Co. (Sapporo, Japan), while libraries for mate pairs were constructed by the Long Jump Distance service (Eurofins Genomics GmbH, Ebersberg, Germany).[1,2] After massively parallel sequencing of the libraries, adaptor sequence removal and quality filtering were performed using CutAdapt software[3] with the BWA trimming algorithm and the parameter of Q20 for paired-end sequences. To reduce the mis-assembly rate, potential PCR duplicate reads were filtered out using the mapping information to the AGM genome (see Materials and methods in the main text). After filtering, error correction and constructing contigs were conducted using the pipeline of SGA software,[4] which yielded the longest contig N50 length over other different assembly software for our data (data not shown). The *k*-mer used for error correction was 61 and overlap length parameters for the FM-merge and assemble processes were 65 and 75, respectively. After assembling contigs, they were connected using three mate-pair library sequences of different insert lengths, as well as paired-end sequences (Supplementary Table 1). Contigs longer than 200 bp were used for scaffolding by the SSPACE

software[5] with a minimal link parameter (*k*) of 5 and maximum ratio parameter (*a*) of 0.7. Regarding scaffolding, libraries were added to scaffolding in an ascending manner with respect to the insert size length.

RNA-seq

Total RNA was extracted from Vero JCRB0111 cells, and purified on RNeasy spin columns (Qiagen) according to the manufacturer's instructions. The mRNA fraction was enriched with the FastTrack MAG micro mRNA isolation kit (Invitrogen), according to the manufacturer's instructions. A cDNA library was then synthesized from 10 ng of the enriched mRNA with the ScriptSeq™ v2 RNA-Seq Library Preparation Kit (Epicentre, Biotechnologies, Madison, WI) with 15 cycles of amplification according to the manufacturer's instructions. The RNA-seq library was electrophoresed on 1% agarose gel and the library with a selected size range (250–700 bp) having specific adapters was purified with the Wizard® SV Gel and PCR Clean-Up System (Promega). The RNA-seq library was subjected to 81–mer paired–end sequencing with Genome Analyzer IIx (Illumina Inc) for whole-transcriptome sequencing. Sequencing reads obtained were 20,104,436 pairs.

Phylogenetic analysis

For phylogenetic analysis of mitochondrial genomes of the Vero cell line, four *Chlorocebus* species, and *Macaca mulatta* (Genbank accession numbers: NC_007009, NC_009747, NC_008066, and NC_009748), the alignment of paired-end reads to *C. sabaeus* was used to generate a consensus sequence of the Vero cell line mitochondrial genome. Whole mitochondrial genomes were analyzed using MEGA6 software.[6] The MUSCLE algorithm[7] and Neighbor-joining method[8] with Kimura’s 2-parameter distance[9] were used for alignment and tree reconstruction, respectively.

Detection of genomic rearrangements in the Vero JCRB0111 cell line

Although we selected candidate regions that were supported by multiple split reads, these junction sequences were not present in the draft assembly sequence of AGM due to the incompleteness of the draft genome. Therefore, we searched the junction sequences against the draft genome sequences of AGM and rhesus macaque (rheMac2) using BWA, and filtered out the candidate regions when the predicted junction sequences were mapped on the draft genome sequences across the boundaries.

It may be noteworthy that a total of 651 deletions, 126 duplications, 103 inversions, and 312 translocations were putatively identified by a bioinformatic analysis with loose criteria (number of paired-end support and split-read ≥ 5); however, many of the putative translocation sites were not validated by PCR analysis with primer sets striding the putative breakpoints, which suggested that the loose criteria may have produced many false-positive sites.

PCR and Sanger sequencing analyses of chromosomal deletions

The genomic DNAs of Vero JCRB0111 cells, Vero ATCC (CCL81) cells, and PBMC were isolated with the Blood Genomic DNA Extraction Mini Kit (Favorgen, Ping-Tung, Taiwan). Primers were designed based on information of the predicted junction regions (Supplementary Table 6). PCR was performed with PrimeSTAR GXL (Takara Bio, Ohtsu, Japan) on equal amounts of genomic DNA. The PCR products were separated by agarose gel electrophoresis, and stained with ethidium bromide, using the 1-Kb Plus DNA Ladder (Life Technology) as a molecular marker. When PCR products were detected, they were also subjected to Sanger sequencing with the ABI3100 Genetic Analyzer (Applied Biosystems).

Characterization of proviral simian type D retrovirus (SRV) in the Vero JCRB0111 genome

To identify nucleotide variations and the redundancy of endogenous SRV, SRV-related short reads from all paired-end short reads (insert size avg.: 317 bp) of the Vero JCRB0111 cell line were

collected using the Burrows-Wheeler Aligner (BWA) Smith-Waterman alignment (SW) mapping technique[10,11] with 15 complete genome sequences of SRV as reference sequences. The short reads (0.025%) obtained were assembled by platanus v1.21, followed by PRICE[12] extending at a 100% identity cut-off and gap-closing between contigs. A single reasonable contig (9.2 kb) was obtained, followed by gene assignment and LTR finding analysis, which suggested that the 8367 bp complete SRV genome sequence was identified. All the short reads obtained were remapped to the SRV-Vero genome sequence by BWA-SW mapping, followed by the extraction of genetic variations using the Sequence Alignment/Map program (SAMtools)[11].

References associate with supplementary materials and methods

1. Williams, L.J., Tabbaa, D.G., Li, N. *et al.* 2012, Paired-end sequencing of Fosmid libraries by Illumina, *Genome Res.*, **22**, 2241-2249.
2. Naito, K., Kaga, A., Tomooka, N. and Kawase, M. 2013, De novo assembly of the complete organelle genome sequences of azuki bean (*Vigna angularis*) using next-generation sequencers, *Breed Sci*, **63**, 176-182.
3. Martin, M. 2011, Cutadapt removes adapter sequences from high-throughput sequencing reads, *EMBNet J*, **17**.
4. Simpson, J.T. and Durbin, R. 2012, Efficient de novo assembly of large genomes using compressed data structures, *Genome Res.*, **22**, 549-556.
5. Boetzer, M., Henkel, C.V., Jansen, H.J., Butler, D. and Pirovano, W. 2011, Scaffolding pre-assembled contigs using SSPACE, *Bioinformatics*, **27**, 578-579.
6. Tamura, K., Stecher, G., Peterson, D., Filipski, A. and Kumar, S. 2013, MEGA6: Molecular Evolutionary Genetics Analysis version 6.0, *Mol. Biol. Evol.*, **30**, 2725-2729.
7. Edgar, R.C. 2004, MUSCLE: multiple sequence alignment with high accuracy and high throughput, *Nucleic Acids Res.*, **32**, 1792-1797.
8. Saitou, N. and Nei, M. 1987, The neighbor-joining method: a new method for reconstructing phylogenetic trees, *Mol. Biol. Evol.*, **4**, 406-425.
9. Kimura, M. 1980, A simple method for estimating evolutionary rates of base substitutions through comparative studies of nucleotide sequences, *J. Mol. Evol.*, **16**, 111-120.
10. Li, H. and Durbin, R. 2010, Fast and accurate long-read alignment with Burrows-Wheeler transform, *Bioinformatics*, **26**, 589-595.
11. Li, H., Handsaker, B., Wysoker, A. *et al.* 2009, The Sequence Alignment/Map format and SAMtools, *Bioinformatics*, **25**, 2078-2079.
12. Ruby, J.G., Bellare, P. and Derisi, J.L. 2013, PRICE: software for the targeted assembly of

components of (Meta) genomic sequence data, *G3*, **3**, 865-880.

Legends to supplementary figures

Figure S1. Karyotyping of AGM PBMC and Vero cells. (*A*) The chromosome number in female AGF PBMC based on 100 Giemsa-stained metaphases showed that the modal number was 60 chromosomes. The loss or gain of one chromosome detected in two cells occurred during the cell culture for 11 days. (*B*) G-banded karyotype of AGM PBMC, showing 2n=60, XX. (*C,D*) Examples of M-FISH on Vero metaphases. Although these two cells had 59 chromosomes, der(7) in these karyotypes differed from the main clone shown in Fig. 1*D* and der(14) was duplicated in *D*.

Figure S2. Sequence analyses of genomic deletions in Vero cells. (*A*) Sequencing of PCR amplicons are shown in Fig. 3. The PCR reaction mixtures of Vero JCRB0111 and AGM-PBMC were used as templates. Underlined primer names indicate the primers used in the sequencing reaction. Note that the sequence chromatogram of “chromosome13” in AGM-PBMC contained 2 sequences, which was consistent with the PCR analysis. (*B*) Predicted models of genomic alleles in AGM-PBMC and Vero cells.

Figure S3. Distribution of heterozygous single nucleotide variants (SNV) density and the homozygous to heterozygous SNV ratio in the 1Mb-size window. The lighter area represents the higher density of windows. The distribution was bimodal and the lower left peak corresponded to potential loss-of-heterozygosity (LOH) regions. On the basis of the distribution, the cut-off criteria were set as follows: heterozygosity < 0.0005 and the homozygous to heterozygous SNV ratio < 0.2.

Table S1. Summary of genome-sequencing libraries of Vero JCRB0111 cells.

Library	median insert size[a]	Read length	Read number (million)[b]	Scaffold N50 (kb)
Pair-end	302	101	2,554	16.6
Mate-pair #1	1606	101	261	37.7
Mate-pair #2	7206	101	74	314.0
Mate-pair #3	18342	101	56	507.9

a) estimated in the scaffolding process
b) number of reads after the quality filter

Table S2. Comparison of mitochondrial genome sequences between the Vero cell line and *Chlorocebus* species.

Species name	Genbank acc.	match	mismatch	divergence
Chlorocebus aethiops	NC_007009.1	15169	891	0.055
Chlorocebus pygerythrus	NC_009747.1	15249	951	0.059
Chlorocebus sabaeus	NC_008066.1	16120	108	0.007
Chlorocebus tantalus	NC_009748.1	15280	903	0.056

Table S3. Number of SNVs and small indels in the Vero cell line genome using the rhesus macaque genome sequence as a reference.

	SNVs	Deletions	Insertions
Nonsynonymous	101,893/32,384	–	–
Synonymous	159,454/28,168	1,031/249	889/114
Nonsense	1,334/969	1,492/578	1,388/261
Untranslated region	199,398/33,699	12,595/1891	12,322/1065
Intron	1,616,4215/1,967,661	875,631/109,769	845,617/63,083
Intergenic	34,541,805/5,283,726	1,728,114/253,188	1,674,883/143,782
Total	51,168,099/7,346,607	2,618,863/365,675	2,535,049/208,305

* Homozygous and heterozygous changes are shown to the left and right of the slashes, respectively. Frameshifting and non-frameshifting indels were classified into nonsense and synonymous categories, respectively.

Table S4. Summary of large deletions in the Vero JCRB0111 cell genome.

Accession	Chromosome	Strand	txStart	txEnd	Exon count	Gene name
AGM position	chr12_50661238:8852739					
MMU position	chr15:47636646-56737738					
XM_002800070.1	chr15	-	47877989	47892767	2	LOC100430678
XM_001104190.2	chr15	+	47894478	49163487	7	LINGO2
XM_002800072.1	chr15	+	49521355	49548196	11	C15H9orf72
XM_001104813.2	chr15	+	49565881	49768793	4	LOC706919
XM_001104958.2	chr15	-	49570708	49571340	1	LOC707234
XM_002800073.1	chr15	+	49697993	49698730	2	LOC100423218
XM_001105128.2	chr15	+	49798331	49810364	8	C15H9orf11
XM_001105270.2	chr15	-	49863954	49985846	23	TEK
XM_001105415.2	chr15	-	50030368	50148315	20	IFT74
XM_001105486.2	chr15	+	50093629	50108941	5	LRRC19
XM_001105698.2	chr15	+	50156501	50198501	14	PLAA
XM_001106045.2	chr15	+	50210238	50263005	6	C15H9orf82
XM_001106111.2	chr15	+	51393196	51394111	1	CCDC89
XM_001106373.2	chr15	+	53236498	53371636	7	ELAVL2
XM_001106627.2	chr15	-	54599731	54604567	2	DMRTA1
XM_001097962.2	chr15	+	54650989	54780607	2	LOC709444
XM_001106881.2	chr15	-	54967969	54986461	2	HNRPA1
XM_001107263.2	chr15	+	55028382	55033946	2	CDKN2B
XM_001098554.2	chr15	+	55060775	55068354	4	CDKN2A
XM_001107077.2	chr15	-	55157607	55210950	8	MTAP
XM_001107329.2	chr15	-	55567555	55568427	1	LOC709559
XM_001107458.2	chr15	-	55604365	55604935	1	IFNA8
NM_001135794.1	chr15	+	55637002	55637569	1	IFNA2
XM_001099165.2	chr15	-	55666769	55674326	4	IFNA1 or 13
XM_001099374.2	chr15	+	55683719	55684406	2	IFNA6
XM_002800107.1	chr15	+	55702345	55706738	1	LOC710804
XM_001107576.2	chr15	+	55738544	55739315	1	IFNA14
XM_001107635.2	chr15	+	55746743	55747313	1	IFNA4
XM_001107693.2	chr15	+	55756603	55757173	1	LOC710654
XM_001107754.2	chr15	+	55769988	55770558	1	IFNA17
XM_001107817.2	chr15	+	55774329	55774899	1	IFNA4
XM_001107884.2	chr15	+	55778756	55779326	1	IFNA17
XM_001107940.2	chr15	+	55803649	55804219	1	IFNA17
XM_001107999.2	chr15	+	55818498	55819068	1	IFNA17
XM_001108051.2	chr15	+	55822820	55823387	1	IFNA21
XM_001108113.2	chr15	+	55840228	55841195	1	IFNW1
NM_001135795.1	chr15	+	55915846	55916410	1	IFNB1

XM_001099778.2	chr15	+	55962896	55999161	9	KIAA1797
XM_002800120.1	chr15	-	55998516	56316036	42	KIAA1797
XM_001108265.2	chr15	+	56308948	56309811	1	LOC711644
XM_001108646.2	chr15	+	56376264	56658076	10	MLLT3
AGM position	chr21_115702018:572661					
MMU position	chr3:184424652-184999217					
XM_001094652.2	chr3	+	183454124	185713854	24	CNTNAP2
AGM position	chr21_115967186:96000					
MMU position	chr3:184700740-184795247					
XM_001094652.2	chr3	+	183454124	185713854	24	CNTNAP2
AGM position	chr9_79491540:293541					
MMU position	chr9:85591097-85888674					
XM_001099261.2	chr9	-	84959245	86303176	18	PRKG1
AGM position	chr7_39221662:198099					
MMU position	chr5:83835008-84051641					
XM_001095828.2	chr5	+	83083150	83850701	9	CCSER1
XM_001101881.1	chr5	-	83896501	83898179	1	LOC704348
AGM position	chr10_504044:1327					
MMU position	chr13:136225605-136226934					
no gene						
AGM position	chr13_67374655:1268					
MMU position	chr4:102475829-102477104					
XM_001088619.2	intron					AIM1
AGM position	chr15_12462694:1372					
MMU position	chr2:109860749-109862122					
XM_001101914.2	intron					LOC712808
AGM position	chr23_50391086:1316					
MMU position	chr6:144295261-144296573					
no gene						
AGM position	chr2_9021672:1048					
MMU position	chr10:9442183-9443282					
no gene						

Chr, chromosome; txStart/txEnd, transcriptional start/transcriptional end.

Table S5. Single nucleotide mutation and insertion position and mix population rate of the truncated SRV provirus sequence in Vero JCRB0111.

Position	Reference sequence	Variant sequence	Total depth	Reference frequency (%)	Variant frequency (%)	Variant type	Gene	Detected mutation	Amino acid substitution
5671	T	A	6911	75.3	24.7	stop gained	*pol*	tta>tAa	L788stop
6503	C	+A	3945	86.8	9.3	frame-shift	*env*		
7100	C	T	5646	91.8	8.1	stop gained	*env*	cga>Tga	R379stop
7586	C	T	2373	84.7	15.2	stop gained	*env*	cga>Tga	R541stop

Table S6. PCR primers used to validate deletions.

Chr12	s1	CCTTAGCAACATAATTCATGTCAGCCCA
	as1	CTCAGTTTTGGGTTCCTCCTCTGGT
	s2	GTGCCATCCTGAACTCAGAGATTACG
	as2	CAGTCTTAGGCACCTTTGGTTTGCT
Chr21-1	s1	CCTGACGACTCAATGTCCTGCTTC
	as1	TGTTCCACGCTCCAGAATTAGTCCACG
	s2	GCCTGAAAGATGAAAGAATGGAGTCACAG
	as2	CTGGTTCACAAGCACCAACTGTTAAGC
Chr21-2	s1	CCTGTCGGTAGATCAAGGTGTTGAG
	as1	CTCCTTTAACAAGACCAGTCTCTTCACAG
	s2	CAAGCAGGAACTGAACTGTGAGCTAAG
	as2	GAATGCTGCAGATGACTCCTGTATTCC
Chr9	s1	GTCAGGATCATTTGAGGAGATAGGCTTTG
	as1	CTGAGTCACAGAACTTGGACATCTAGTC
	s2	CAAGAGGTTAAAGAGAGCCCTTAGAGATTC
	as2	GAGTTATGTGCGGAGACCTGAAGAC
Chr7	s1	CCGAGAAGTCAGCTGTTAGTCTGATG
	as1	GCCACAGTCAGGAATGACATATTGGC
	s2	GTGGGAAAGACATTAAGGCACTGTGG
	as2	GAGGGAGAAGAAAAACACAACCTGG
Chr1	s1	ATCTCACAGGGAAAGCTGCCACC
	as1	TGGAATTGGGGTTAAACGGGTTTGAAGG
Chr10	s1	TCCTCCCTGGTACTCTGTCCTG
	as1	CCATTCCAGTAGGCACACACAGTG
Chr13	s1	TAGGGACGCTGGCCTTCAGAG
	as1	GTTCTATCACAACCTTTGCCCCTATGAC
Chr15	s1	TAGACAAGCTCCCTTTGTACTGGTCAC
	as1	TGGTTCCGAACTCCTAAGCTCAAGTG

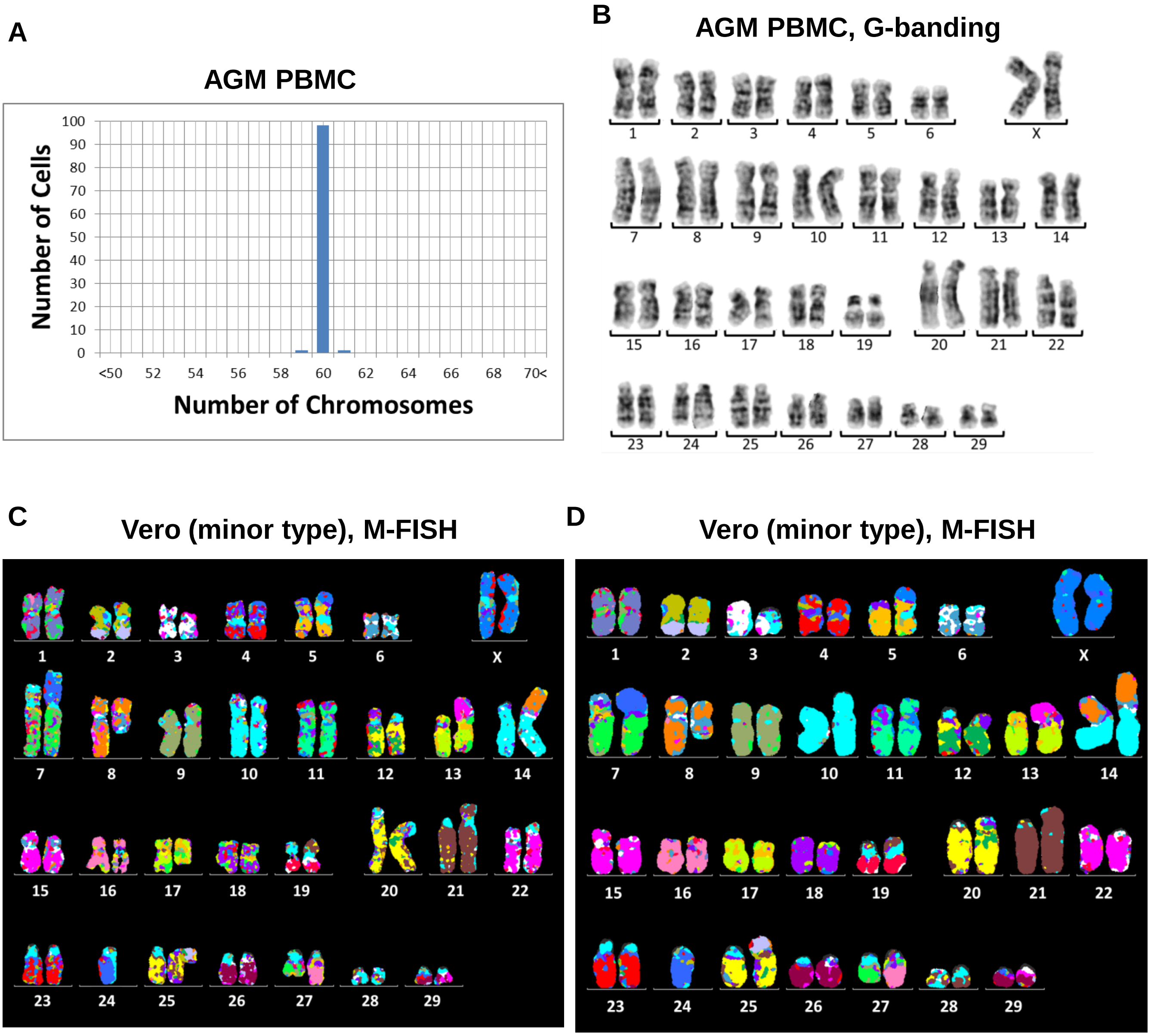

Supplementary Figure 1

A

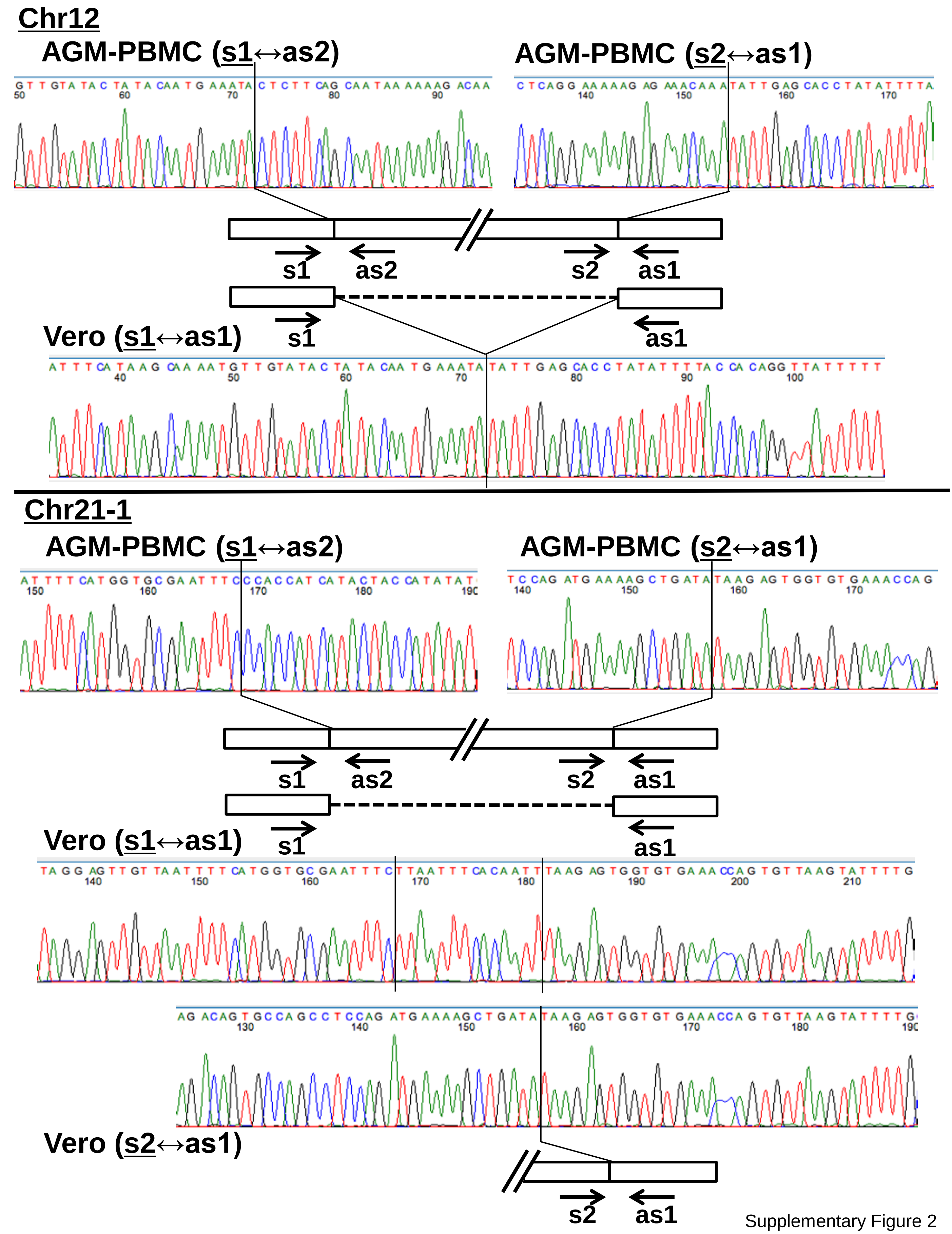

Supplementary Figure 2

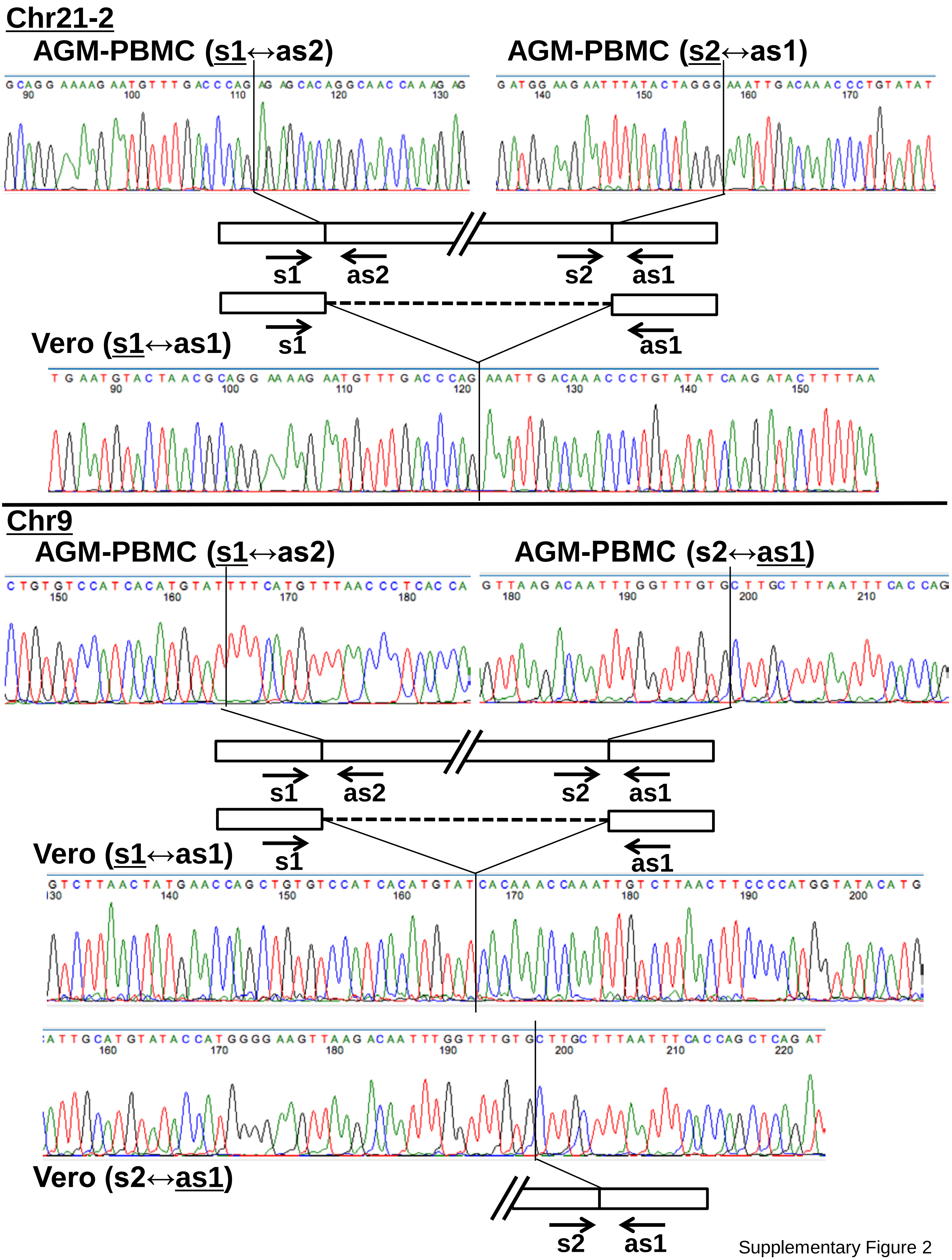

Supplementary Figure 2

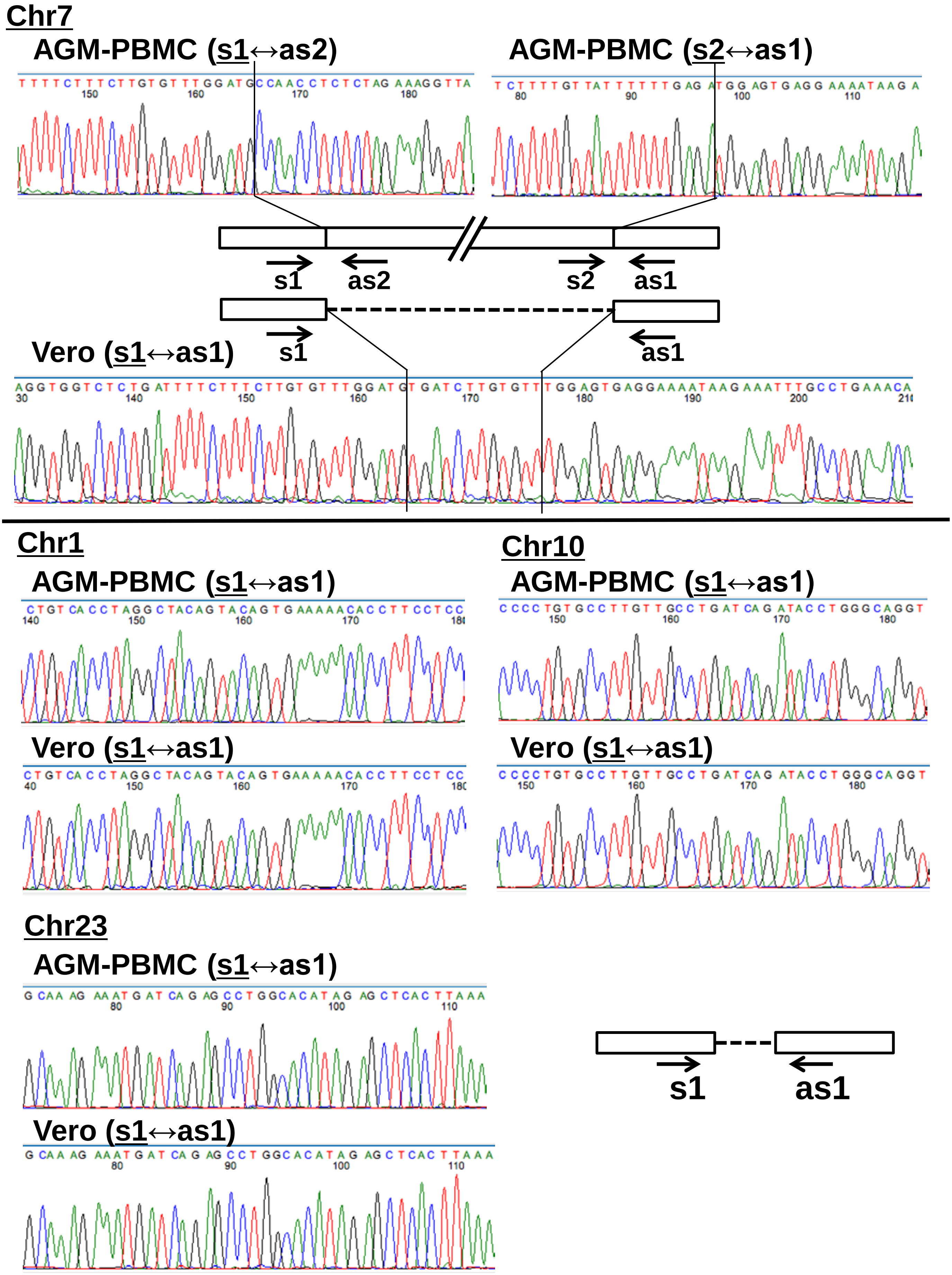

Supplementary Figure 2

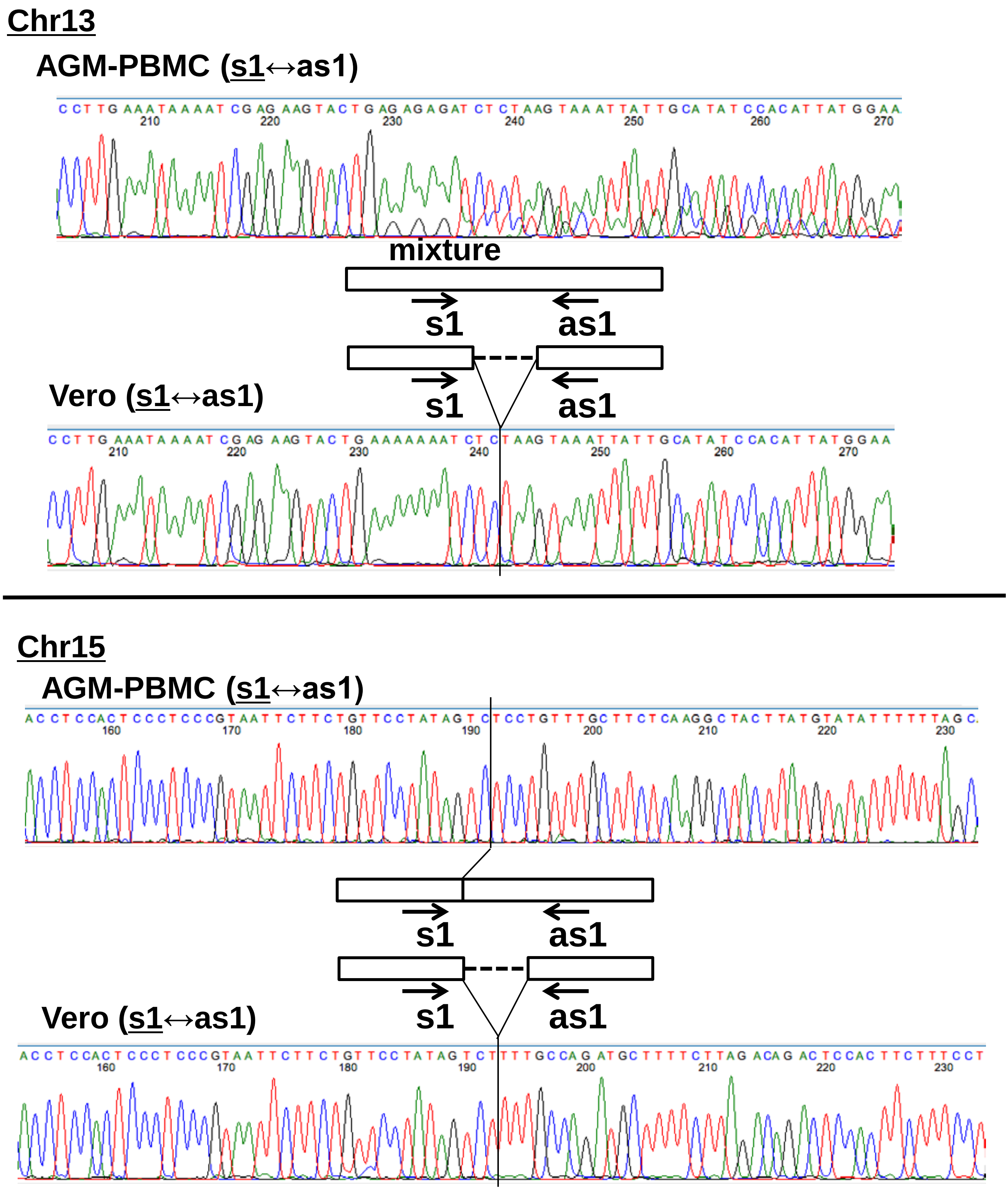

Supplementary Figure 2

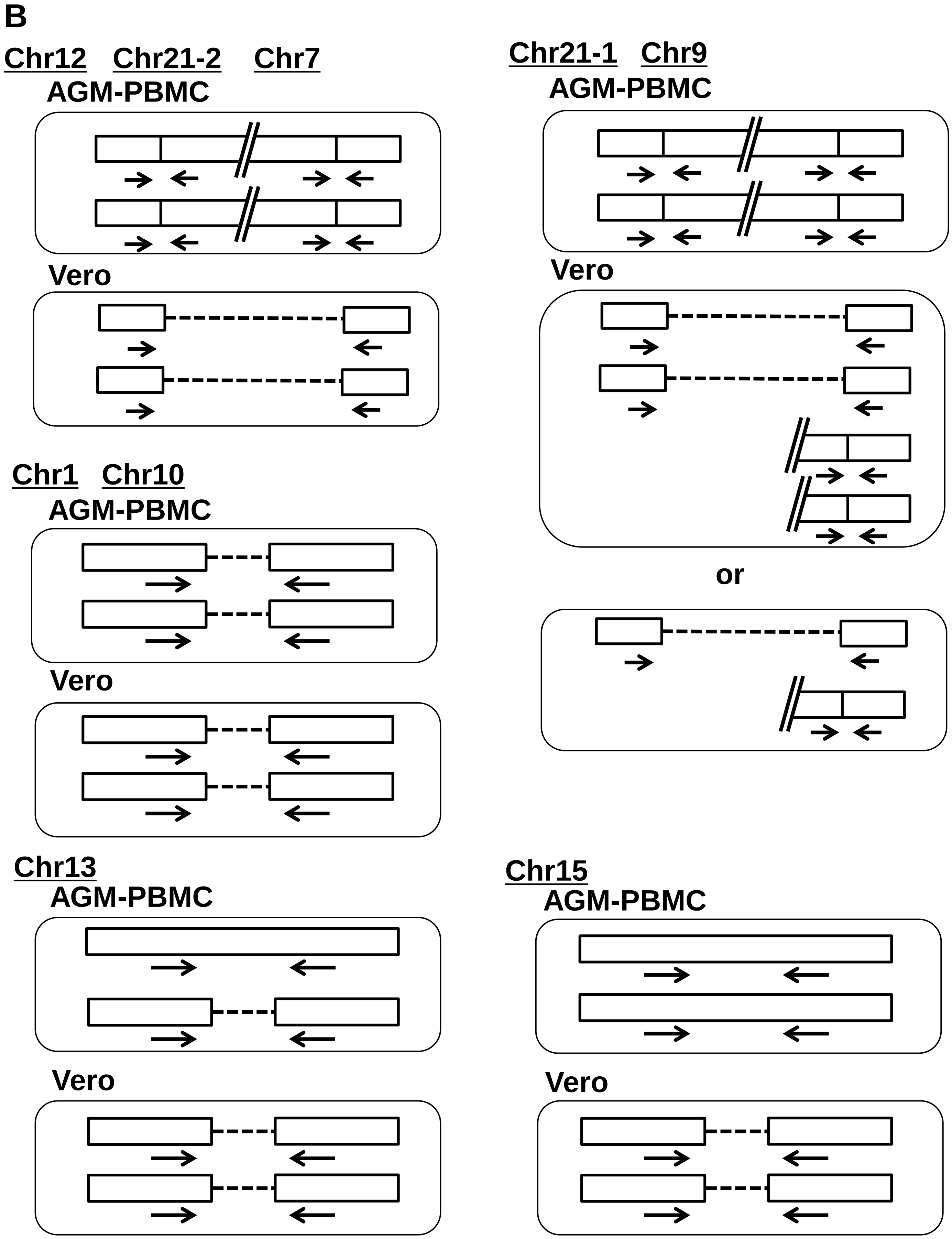
B
Chr12
Chr21-2
Chr7
AGM-PBMC
Vero
Chr21-1
Chr9
AGM-PBMC
Vero
or
Chr1
Chr10
AGM-PBMC
Vero
Chr13
AGM-PBMC
Vero
Chr15
AGM-PBMC
Vero

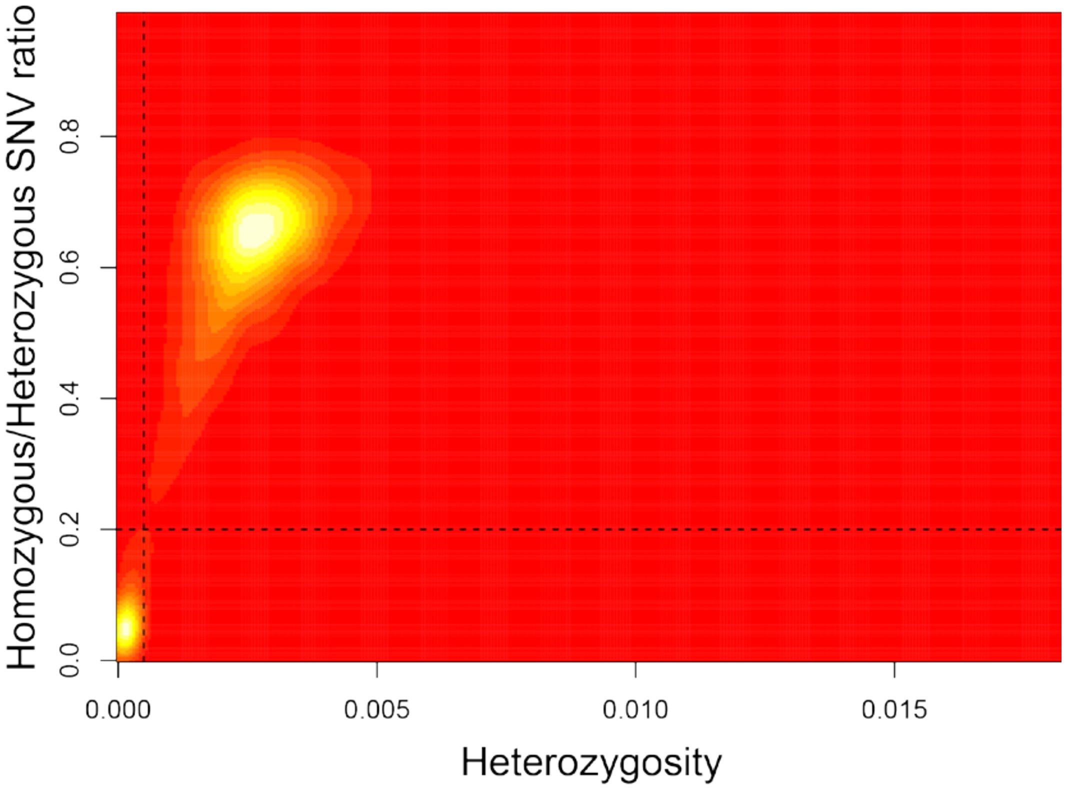

Supplementary Figure 3
